# A novel method for site-specific chemical SUMOylation: SUMOylation of Hsp90 modulates co-chaperone binding *in vitro*

**DOI:** 10.1515/hsz-2018-0251

**Published:** 2019-04-24

**Authors:** Annemarie Wolmarans, Allison Kwantes, Paul LaPointe

**Affiliations:** Department of Cell Biology, Faculty of Medicine and Dentistry, University of Alberta, Edmonton T6G 2H7, Alberta, Canada; Department of Biology, The King’s University, Edmonton T6B 2H3, Alberta, Canada

**Keywords:** Aha1, ATPase, crosslinking, Hsp90, Smt3, SUMO

## Abstract

SUMO is covalently attached to lysine side chains in target proteins by the action of a cascade of E1, E2, and E3 ligases. Unlike ubiquitin, SUMO does not target proteins for degradation but rather plays a regulatory role in activating target proteins or directing them to multiprotein complexes. Isolating SUMOylated proteins from native sources is challenging because of the low stoichiometry of SUMOylation that occurs for any given target protein in cells. Here we report a novel strategy to couple SUMO to the site of a target lysine for the purpose of *in vitro* study. Introduction of a single cysteine after the *C* terminal diglycine motif and a cysteine in place of a target lysine in a substrate protein allows for efficient and specific crosslinking of SUMO using a homo-bifunctional maleimide crosslinker. We demonstrate that SUMO can be crosslinked in this manner to amino acid position 178 in the dimeric molecular chaperone, Hsp90. Chemically SUMOylated Hsp90 has very similar ATPase activity compared to unmodified Hsp90 but displays preferential co-chaperone binding *in vivo*. Our novel strategy can easily be applied to other SUMOylated or ubiquitinated target protein *in vitro*.

## Introduction

Since its discovery in 1996, the small ubiquitin-like modifier, SUMO, has been the focus of intensive study (Matunis et al., 1996). A myriad of proteins involved in a diverse array of pathways, from signal transduction (Zhao, 2007) to the cell cycle (Eifler and Vertegaal, 2015) to DNA repair (Dantuma and van Attikum, 2016), are regulated by SUMOylation.

SUMO is synthesized as a pro-protein that is activated when proteases remove a short peptide from the *C* terminus of the protein to reveal a di-glycine motif (Hay, 2007). The same proteases (ubiquitin-like protein-specific proteases, ULPs, in yeast; sentrin/SUMO-specific proteases, SENPs, in mammals) that generate mature SUMO are also responsible for removal of SUMO from target proteins (Mukhopadhyay and Dasso, 2007). Conjugation of SUMO onto lysine side chains in target proteins is mediated by similar machinery responsible for the conjugation of ubiquitin (Gareau and Lima, 2010). The action of E1 and E2 enzymes (and sometimes an E3 ligase) drive the conjugation of SUMO to target proteins that can then interact with other proteins, many of which contain SUMO interacting motifs (SIMs) (Minty et al., 2000; Hecker et al., 2006).

While the mechanism by which proteins become SUMOylated is known, the manner in which SUMOylation alters the function of target proteins is not well understood. This is in part due to the difficulty of generating SUMOylated proteins for *in vitro* study. Isolation of SUMOylated proteins from native sources is difficult because of the apparently low stoichiometry of the modification and often the presence of multiple target sites (Nie et al., 2015). Overexpression of the SUMO pro-protein can shift the proportion of SUMOylated proteins in a cell, presumably by competing with processing enzymes like Ulp1p and Ulp2p (which are responsible for both generating mature SUMO as well as removing SUMO from target proteins) (Mollapour et al., 2014). In-frame insertion of the SUMO coding sequence in target proteins is a strategy that has also been employed with some success (Babic et al., 2006; Tan et al., 2008). Alternatively, protein SUMOylation can be carried out *in vitro* with purified ligases but this does not allow for targeting of specific sites (Macauley et al., 2006; Vethantham and Manley, 2009). Consequently, generating large amounts of protein that is SUMOylated in a site-specific manner for *in vitro* study remains a challenge.

Many proteins are SUMOylated in response to cell stress (Guo and Henley, 2014; Enserink, 2015) and several stress proteins have been linked to modification of substrates (Brunet Simioni et al., 2009; Yang et al., 2013). Interestingly, the abundant molecular chaperone, heat shock protein 90 (Hsp90), has recently been identified as a target for SUMOylation (Mollapour et al., 2014). Hsp90 is a dimeric ATPase that acts on hundreds of substrate proteins called ‘clients’. These client proteins include central players in almost all biological pathways (Schopf et al., 2017). To activate clients, Hsp90 functions in the context of a conformationally dynamic ATPase cycle. Progression through this functional cycle is guided by the sequential interaction of co-chaperones which are vastly less abundant than Hsp90 itself (Ghaemmaghami et al., 2003). Post-translational modifications (PTMs) in Hsp90 are thought to selectively recruit co-chaperones, or modulate the conformational consequences of their binding, and modulate ATP hydrolysis during client activation (Scroggins et al., 2007; Mollapour et al., 2010, 2011, 2014; Mollapour and Neckers, 2012; Solier et al., 2012; Soroka et al., 2012; Wang et al., 2012; Xu et al., 2012; Woodford et al., 2016). How PTMs modulate the recruitment and dwell time of specific co-chaperones has not been fully elucidated and this is further complicated by PTMs in co-chaperones themselves. For example, the co-chaperone cdc37, that is responsible for delivering client kinases to Hsp90 for maturation, is phosphorylated at serine 13 which is critical for its function (Miyata and Nishida, 2004). Another example is Aha1 which is phosphorylated by c-Abl at tyrosine 223 to promote its recruitment to Hsp90 (Dunn et al., 2015). Overall, PTMs play a central role in coordinating progression through the Hsp90 client maturation cycle.

The effect of SUMOylation on Hsp90 function has been inferred from studies in cells in which SUMOylation of lysine 178 (or K191 in human Hsp90) is blocked (by substitution with arginine) or generally enhanced (by overexpression of SUMO) (Mollapour et al., 2014). SUMOylation of Hsp82p at this site appears to limit chaperone activity towards clients like the cystic fibrosis transmembrane conductance regulator (CFTR) and the glucocorticoid hormone receptor (GR). In cells where SUMOylation of lysine 178 is enhanced, more Aha1p is recovered in complex with Hsp90 while the amounts of cdc37p, Sba1p and Sti1p remain the same. Conversely, cdc37p, Sba1p, and Sti1p, are recovered in complex with Hsp82p^K178R^, but Aha1p is not.

Mechanistic interpretations of Hsp90 complex recovery from cells is made difficult by the dynamic nature of co-chaperone exchange and the involvement of numerous other PTMs that likely occur in coordination with modifications like SUMOylation. How SUMOylation affects enzymatic properties like ATPase activity cannot be determined without a means of generating purified protein that is quantitatively modified at a specific site. We have devised a strategy to covalently link the yeast SUMO, Smt3p, to a specific site in Hsp82p that takes advantage of the fact that both of these proteins natively lack cysteine residues. We constructed an Hsp82p mutant that harbors a cysteine residue in place of the target lysine (K178) and an Smt3p mutant that contains a cysteine downstream of the *C* terminal diglycine motif. Because no other cysteines are present, these proteins can be specifically and efficiently crosslinked to one another using a homo-bifunctional maleimide crosslinker. This chemically SUMOylated Hsp82p retains both intrinsic and stimulated ATPase activity and recapitulates preferential co-chaperone interactions that are observed *in vivo*.

## Results

### Covalent addition of Smt3p to Hsp82p

**Figure 1:**
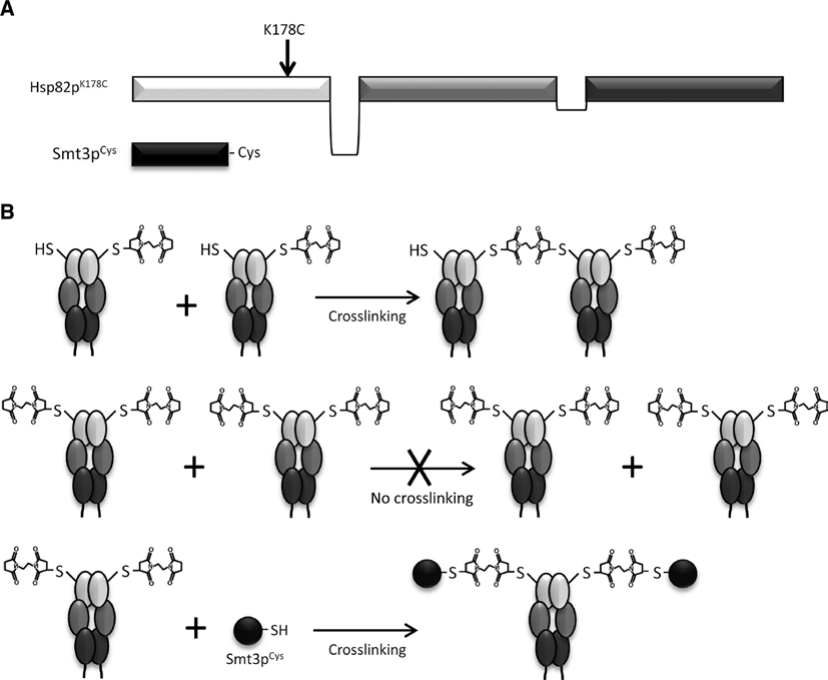
Constructs and crosslinking strategy for the covalent attachment of Smt3p^Cys^ to Hsp82p^K178C^. (A) Full-length Hsp82p construct harboring a cysteine in place of lysine at residue 178, and mature form of Smt3p harboring a cysteine downstream of the diglycine motif. (B) Using the homo-bifunctional maleimide crosslinker, BMOE, singly derivatized Hsp82p^K178C^ will crosslink to itself to form Hsp82p^K178C^-Hsp82p^K178C^ dimers. However, if derivatized with a vast excess of BMOE, every molecule of Hsp82p^K178C^ will be conjugated to BMOE thereby preventing the formation of Hsp82p^K178C^-Hsp82p^K178C^ crosslinked dimers. When all molecules of Hsp82p^K178C^ are derivatized then near-complete crosslinking to Smt3p^Cys^ can be achieved.

**Figure 2:**
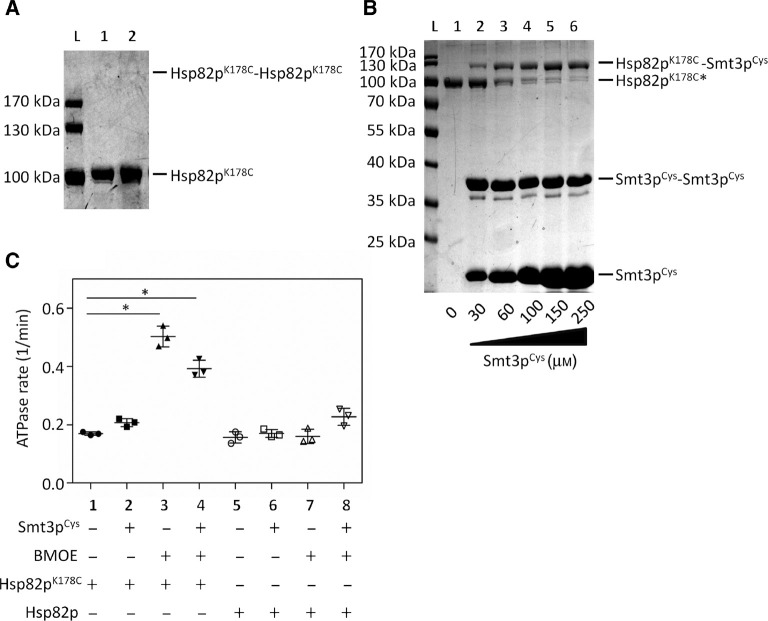
Covalent addition of Smt3p^Cys^ to Hsp82p^K178C^ and ATPase analysis. (A) No significant intermolecular (Hsp82p^K178C^-Hsp82p^K178C^) crosslinking was observed in the presence of BMOE. Crosslinking reactions contained 32 μm Hsp82p^K178C^ with DMSO (lane 1) or 200 μm BMOE (lane 2) that was incubated for 1 h at room temperature. Ten microliters of the 40 μl crosslinking reactions were visualized on an 8% sodium dodecyl sulfate (SDS) gel stained with Coomassie blue. (B) Maximal crosslinking of Smt3p^Cys^ to Hsp82p^K178C^ occurred upon the addition of 250 μm Smt3p^Cys^. Hsp82p^K178C^ was derivatized (*) in the presence of 200 μm BMOE for an hour prior to incubating with Smt3p^Cys^. To crosslink Smt3p^Cys^ to Hsp82p^K178C^, 2 μm derivatized Hsp82p^K178C^ was incubated with 0, 30, 60, 100, 150 or 250 μm Smt3p^Cys^ for an hour at room temperature, followed by quenching the reactions with 30 mm DTT. Ten microliters of the 40 μl crosslinking reactions were visualized on a 12% SDS-polyacrylamide gel electrophoresis (PAGE) gel stained with Coomassie blue. These experiments were conducted 6 times (n=6) and a representative gel of the crosslinking reactions are shown in (A) and (B). (C) The intrinsic ATPase activity of Hsp82p^K178C^ is similar to wildtype Hsp82p with and without Smt3p^Cys^ (reactions 1, 2, 5, and 6). A statistically significant [*one-way analysis of variance (ANOVA), *p*<0.5] increase in the intrinsic ATPase rate is observed when Hsp82p^K178C^ is derivatized and conjugated to Smt3p^Cys^). Final ATPase reactions contained 2 μm Hsp82p^K178C^ or wildtype Hsp82p in the presence of 12.35 μm BMOE and/or 100 μm Smt3p^Cys^ where indicated. DMSO and gel filtration buffer were used as controls in reactions not containing BMOE (1, 2, 5, and 6) and Smt3p^Cys^ (1, 3, 5, and 7), respectively. This experiment was carried out 3 times (n=3) in triplicate. Each scatter plot shows the average value and standard deviation.

We constructed a plasmid encoding the processed/mature form of Smt3p harboring an *N* terminal 6×His tag and a cysteine downstream of the *C* terminal diglycine motif (Smt3p^Cys^) (Figure 1A). We also constructed plasmids encoding *N* terminally 6×His-tagged, full length Hsp82p and Hsp82p^K178C^ (Figure 1A). All of these proteins were expressed and purified from bacteria. We selected the short-arm homo-bifunctional maleimide crosslinker bismaleimidoethane (BMOE) because it had the shortest distance (8 Å) between the two reactive maleimide groups. Because we wanted to achieve crosslinking of Hsp82p^K178C^ to Smt3p^Cys^ and not to itself, we first tested Hsp82p^K178C^ alone in a crosslinking reaction containing the recommended concentration of BMOE. Two cysteines can only be crosslinked to one another by a single molecule of BMOE, but not if each cysteine reacts with a separate molecule of BMOE (Figure 1B). We reasoned that, owing to the >6-fold excess of BMOE compared to Hsp82p^K178C^, most, if not all, of the cysteines would react with an individual molecule of BMOE without becoming crosslinked to one another. At a concentration of 32 μm Hsp82p^K178C^ and 200 μm BMOE, we observed only a faint band corresponding to a dimer of Hsp82p^K178C^, suggesting that very few intermolecular crosslinks between Hsp82p^K178C^ proteins occurred (Figure 2A). Based on this, we concluded that each molecule of Hsp82p^K178C^ was effectively derivatized by BMOE before intermolecular crosslinking could take place. If this were the case, addition of a Smt3p^Cys^ would couple with the unreacted end of the BMOE present on each molecule of Hsp82p^K178C^. To test this, we repeated the incubation of Hsp82p^K178C^ with BMOE and subsequently added increasing amounts of Smt3p^Cys^. At low concentrations of Smt3p^Cys^ (30 μm), we observed a prominent band corresponding to a dimer of Smt3p^Cys^ but when increasing concentrations of Smt3p^Cys^ were added (60, 100, 150, 250 μm) we observed a shift of Hsp82p^K178C^ to a higher molecular weight corresponding to Hsp82p crosslinked to Smt3p^Cys^ (Figure 2B). We observed maximal crosslinking of Smt3p^Cys^ to Hsp82p^K178C^ upon the addition of 250 μm Smt3p^Cys^.

### Nucleotides affect accessibility of the side chain at position 178 in Hsp82p

SUMOylation of lysine 178 in cells appears to be dependent on nucleotide binding in Hsp90 (Mollapour et al., 2014). In the original study, Hsp90 mutants that were unable to bind to ATP (Hsp82p^D79N^) were not SUMOylated very efficiently compared to an Hsp90 mutant that could bind but not hydrolyze ATP (Hsp82p^E33A^). To explore this nucleotide dependence in our system we carried out the derivatization and Smt3p^Cys^ crosslinking steps in the presence of no nucleotide, ADP, or the non-hydrolyzable ATP analog, AMPPnP. Crosslinking efficiency of Smt3p^Cys^ was less robust and less efficient (as a function of time) in the presence of ADP than either AMPPnP or in the absence of nucleotide (Supplementary Figures S1 and S2). This suggests that ADP binding promotes the acquisition of a conformational state in Hsp90 where the side chain at position 178 is less exposed.

### ATPase analysis of SUMOylated Hsp82p

We next wondered what effect the K178C substitution, BMOE crosslinker, or Smt3p^Cys^ might have on the ATPase rate of Hsp90 on their own. We therefore measured the ATPase rate of Hsp82p^K178C^ (black, plots 1–4) and wildtype Hsp82p (white, plots 5–8), with and without Smt3p^Cys^, both before and after derivatization with BMOE (Figure 2C). The ATPase activity of the Hsp82p^K178C^ mutant was very similar to that of the wildtype Hsp82p (compare plot 1 to plot 5). The addition of free Smt3p^Cys^ did not significantly affect the ATPase rate of either form of Hsp82p (plot 2 and 6) suggesting that this protein did not influence ATPase activity simply by being present in the reaction. The addition of the BMOE crosslinker resulted in a small but statistically significant increase in the ATPase activity of to Hsp82p^K178C^ (plot 3) but not wildtype Hsp82p (plot 7). This is consistent with only Hsp82p^K178C^ having a cysteine capable of reacting with BMOE. It has been previously shown that the introduction of hydrophobic amino acids near the dimerization interface of Hsp82p results in an enhancement of *N* terminal dimerization and a concomitant increase in ATPase activity (Hawle et al., 2006; Vaughan et al., 2009). The BMOE crosslinker is hydrophobic and could have a similar effect when coupled near the dimerization interface of the Hsp82p *N* domains. The addition of Smt3p^Cys^ to BMOE-derivatized Hsp82p^K178C^ resulted in a small reduction in ATPase activity (plot 4) compared to BMOE-derivatized Hsp82p^K178C^ (plot 3), likely due to the hydrophobic BMOE group being partially buried by Smt3p^Cys^ after crosslinking. There was no statistically significant change in ATPase activity in wildtype Hsp82p when both BMOE and Smt3p^Cys^ were added (plot 8). These data show that the specific crosslinking of Smt3p^Cys^ to position 178 in Hsp82p mildly enhances ATPase activity of the Hsp90 chaperone, possibly due to weak enhancement of *N* terminal dimerization mediated by the hydrophobic BMOE group.

**Figure 3:**
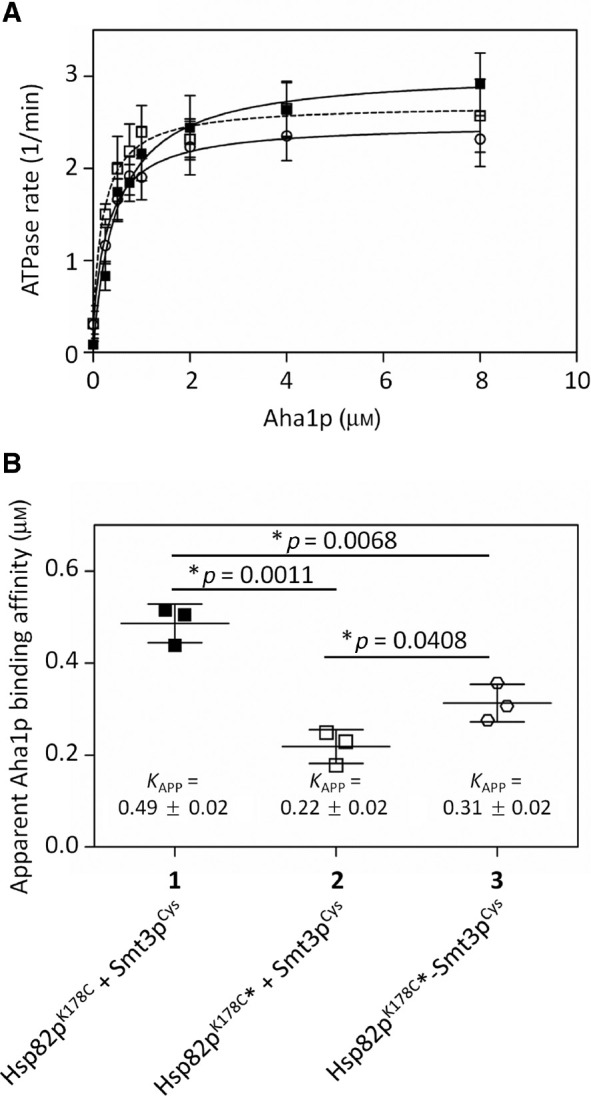
Aha1p-mediated stimulation of SUMOylated Hsp82p. (A) Aha1p titration into non-SUMOylated Hsp82p, derivatized but non-SUMOylated Hsp82p, and SUMOylated Hsp82p. All reactions contained 1 μm non-SUMOylated Hsp82p^K178C^, either treated with DMSO [Hsp82p^K178C^ + Smt3p^Cys^ (1; black squares)] or derivatized with BMOE and quenched with DTT prior to Smt3p^Cys^ addition [Hsp82p^K178C*^+Smt3p^Cys^ (2; white squares)], or SUMOylated [Hsp82p^K178C*^-Smt3p^Cys^ (3; white circles)] and the indicated concentration of Aha1p. This experiment was carried out three times (n=3) in triplicate. (B) The apparent binding affinity (*K*_app_) of Aha1p for non-SUMOylated (1), derivatized but not SUMOylated (2), and SUMOylated Hsp82p (3) was calculated to be 0.49±0.02 μm, 0.22±0.02 μm, and 0.31±0.02 μm, respectively. Statistical significance was determined with an unpaired *t*-test between columns. The apparent affinity for Aha1p was calculated from the individual ATPase assays in (A) using Michaelis-Menton kinetic analysis on Prism GraphPad. Each scatter plot shows the average and standard deviation from three experiments (n=3).

Aha1p binds preferentially to *N* terminally dimerized Hsp90 (Retzlaff et al., 2010; Pullen and Bolon, 2011). If the enhancement in intrinsic ATPase activity we observed upon derivatization of Hsp82p^K178C^ with BMOE was due to enhanced *N* terminal dimerization then we would expect Aha1p binding affinity to be enhanced as well. We titrated Aha1p into ATPase reactions containing Hsp82p^K178C^ and Smt3p^Cys^ but no BMOE, Hsp82p^K178C^ that had been derivatized with BMOE but blocked with DTT prior to the addition of Smt3p^Cys^ (to prevent crosslinking of SUMO to Hsp82p), and Hsp82p^K178C^ crosslinked to Smt3p^Cys^ with BMOE (Figure 3A). We calculated the apparent affinity of Aha1p for Hsp82p in each of these experiments (Figure 3B) and found that the affinity of Aha1p was enhanced upon derivatization of Hsp82p^K178C^ with BMOE, and that this enhanced affinity was somewhat diminished upon crosslinking of Smt3p^Cys^ to Hsp82p^K178C^. Importantly, Aha1p does not contain any SIMs so it is unlikely that recruitment of Aha1p to SUMOylated Hsp90 is through a direct interaction with SUMO. Taken together, these data suggest that coupling of the hydrophobic BMOE group to position 178 in Hsp82p weakly enhances *N* terminal dimerization. Neither derivatization with BMOE alone nor crosslinking of Smt3p^Cys^ had a significant effect on the Aha1p-stimulated ATPase rate of Hsp82p^K178C^.

### ATPase activity of Hsp82p is affected equally by symmetric and asymmetric SUMOylation

**Figure 4:**
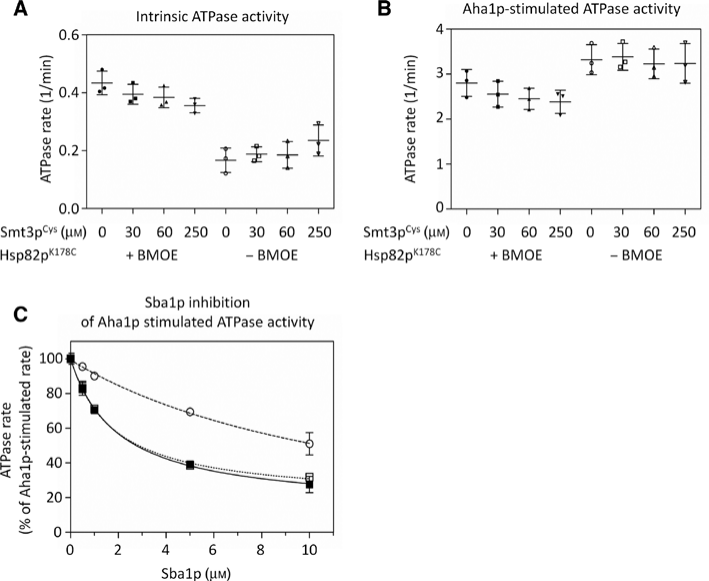
Intrinsic and Aha1p-stimulated ATPase rate of hemi- and dually-SUMOylated Hsp82p and inhibition by Sba1p. The intrinsic (A) and Aha1p-stimulated (B) ATPase activity of derivatized Hsp82p^K178C^ (black) decreases slightly with increased SUMOylation (0–250 μm Smt3p^Cys^). Reactions contained 2 μm derivatized Hsp82p^K178C^ (black) or DMSO-treated Hsp82p^K178C^ (gray), in the absence (A) or presence (B) of 10 μm Aha1p, and 0, 30, 60, or 250 μm (circles, squares, upward-triangles and downward-triangles) of Smt3p^Cys^. The addition of 30, 60 and 250 μm Smt3p^Cys^ corresponds to a 50, 75 >95% band shift from non-SUMOylated to SUMOylated Hsp82p^K178C^ (black). Hemi-SUMOylated Hsp82p refers to a 50% band shift while dually-SUMOylated Hsp82p refers to >95% band shift from non-SUMOylated to SUMOylated Hsp82p. Each scatter plot shows the average and standard deviation from three triplicate experiments (n=3). (C) Sba1p inhibition of the maximally Aha1p-mediated stimulation of non-SUMOylated Hsp82p (black squares), derivatized but non-SUMOylated Hsp82p (white squares), and SUMOylated Hsp82p (white circles). Reactions contained 1 μm Hsp82p^K178C^+Smt3p^Cys^ (black squares), Hsp82p^K178C*^+Smt3p^Cys^ (white squares), or Hsp82p^K178C*^-Smt3p^Cys^ (white circles) with 5 μm Aha1p, and the indicated concentration of co-chaperone Sba1p. Each curve shows the results of three experiments that were each carried out in triplicate (n=3).

The SUMOylation of Hsp90 *in vivo* was reported to occur on only one protomer of the Hsp90 dimer (Mollapour et al., 2014). Because we conjugated Smt3p to both subunits of the dimer in our experiments we wondered if ATPase activity or Aha1p stimulation would be different for asymmetrically SUMOylated Hsp90. To test this, we measured the ATPase activity of Hsp82p^K178C^ that had been derivatized with increasing amounts of Smt3p^Cys^ with and without Aha1p. In our initial crosslinking studies, we observed a concentration-dependent increase in the formation of crosslinked Hsp82p^K178C^-Smt3p^Cys^ conjugates. We reasoned that the proportion of hemi- to dually-SUMOylated Hsp82p dimers would shift accordingly. We tested Hsp82p^K178C^ that had been derivatized with 30, 60, and 250 μm Smt3p^Cys^ to yield mixtures of SUMOylated and non-SUMOylated Hsp82p^K178C^ (54.8%±9.5%, 71.8%±5.9%, and 92.4%±6.1% SUMOylation, respectively). We did not observe any major difference between the intrinsic (Figure 4A) or Aha1p-stimulated ATPase activity (Figure 4B) as the proportion of SUMOylated Hsp90 increased. This suggests that Hsp90 dimers harboring a crosslinked Smt3p^Cys^ on one or both protomers had comparable ATPase activity, both with and without Aha1p-mediated stimulation.

### Aha1p-mediated stimulation of SUMOylated Hsp82p is resistant to inhibition by Sba1p but not Sti1p

Several *in vivo* studies have indicated that stable interaction of Aha1p requires different post-translational modifications of Hsp90 (Mollapour et al., 2010, 2011, 2014). Preventing SUMOylation of Hsp90 by replacing lysine 178 with arginine reduces the recovery of Aha1p in complex with Hsp90 but the mechanism for this is not known (Mollapour et al., 2014). Sba1p binds to the dimerized Hsp90 *N* domains, at or near the site where the Aha1p *C* domain is thought to bind (Koulov et al., 2010; Retzlaff et al., 2010). While both Aha1p and Sba1p are thought to bind to *N* terminally dimerized Hsp90, the optimal Hsp90 conformation for binding to these two co-chaperones is likely different. In support of this, Sba1p can only bind to Hsp90 when in the ATP-bound state whereas Aha1p can bind to nucleotide-free, as well as ADP- and ATP-bound Hsp90, albeit with slightly different affinities (Armstrong et al., 2012). In ATPase reactions Sba1p inhibits Aha1p-mediated stimulation (Harst et al., 2005). We wondered if Sba1p would be less able to inhibit Aha1p-stimulated ATPase activity when Hsp90 is SUMOylated at position 178. We titrated Sba1p into Aha1p-stimulated ATPase reactions containing either SUMOylated (Figure 4C, white circles) or non-SUMOylated Hsp82p^K178C^ (both with and without prior derivatization with BMOE – Figure 4C, white and black squares, respectively). Inhibition of Aha1p-mediated stimulation by Sba1p was greatly reduced when Smt3p^Cys^ was crosslinked to Hsp82p^K178C^ (Figure 4C).

**Figure 5:**
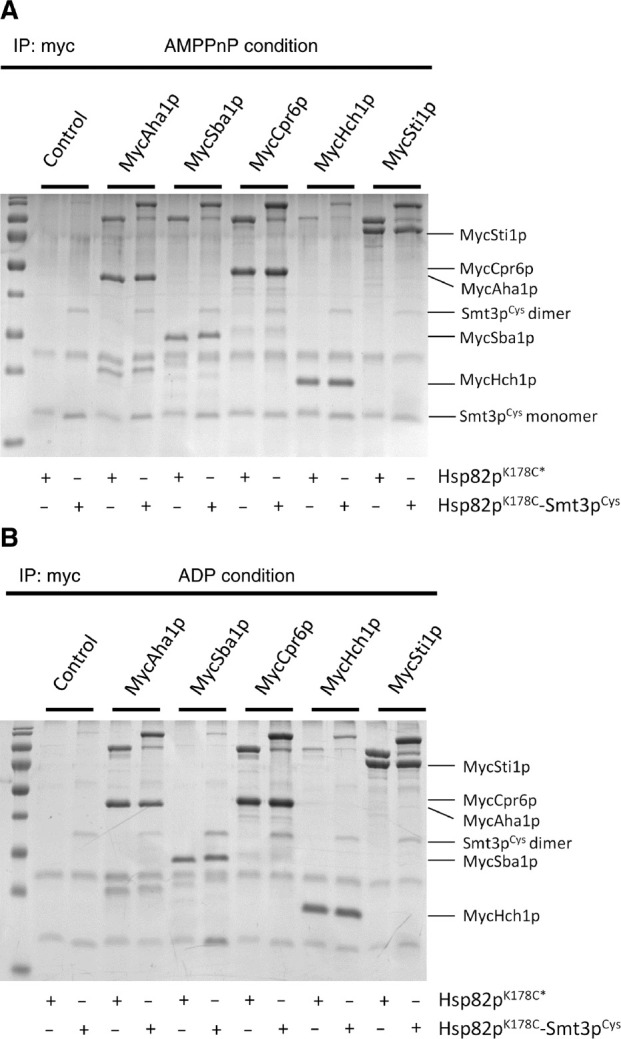
Co-chaperones interact with non-SUMOylated and SUMOylated-Hsp82p equally. Myc-tagged Aha1p, Sba1p, Cpr6p, Hch1p and Sti1p immunoprecipitated non-SUMOylated and SUMOylated Hsp82p^K178C^ to the same extent in both AMPPnP (A) and ADP (B) conditions. Control lanes show the amount of Hsp82p^K178C^ recovered with beads alone, indicating non-specific binding of SUMOylated Hsp82p. Reactions contained 5 μm derivatized Hsp82p (Hsp82p^K178C*^), in the presence or absence of Smt3p^Cys^, which was then quenched with 10 mm DTT for 15 min prior to mixing with 5 μm of Myc-tagged co-chaperones. These 50 μl reactions were incubated for an hour at room temperature. Ten microliters of Ultralink Protein G beads coupled to anti-Myc monoclonal antibodies were added to the 50 μl reactions and then incubated on a rotator at room temperature for 90 min. Beads were then pelleted, washed once in 250 μl of binding buffer, and resuspended in 50 μl SDS sample buffer. Ten microliters of each reaction were run on 12% SDS-PAGE gel and complexes were analyzed by Coomassie blue staining. IP experiments were conducted 3 times (n=3) and representative gels showing the individual reactions are shown in (A) and (B).

To further interrogate the interactions between SUMOylated Hsp90 and co-chaperones we expressed and purified myc-tagged versions of Aha1p, Sba1p and Sti1p, as well as the Aha1p homologue, Hch1p and the TPR co-chaperone Cpr6p. We incubated each of these co-chaperones with Hsp82p^K178C^ and Smt3p^Cys^, both with and without prior crosslinking with BMOE. We carried out these experiments with either AMPPnP or ADP to examine the nucleotide dependence of co-chaperone interaction with SUMOylated and non-SUMOylated Hsp82p. We were able to recover both SUMOylated and non-SUMOylated Hsp82p in complex with all of the myc-tagged co-chaperones we tested (Figure 5). Interesting, both SUMOylated and non-SUMOylated Hsp82p interacted equally well with Sba1p in the presence of AMPPnP. This is in contrast to the apparent reduction in affinity that we observed in our ATPase assays in the presence of Aha1p. SUMOylation may disfavor Sba1p binding only in the presence of Aha1p or only when mixed ATP/ADP-bound states form during ATP hydrolysis (Halpin and Street, 2017). Importantly, we did not recover any free Smt3p^Cys^ or crosslinked dimers of Smt3p^Cys^ in complex with any of the co-chaperones we tested. Therefore, we can rule out the possibility that free or crosslinked dimers of Smt3p are competing with Hsp82p for binding to co-chaperones.

### SUMOylation does not interfere with Aha1p-driven co-chaperone switching *in vitro*

**Figure 6:**
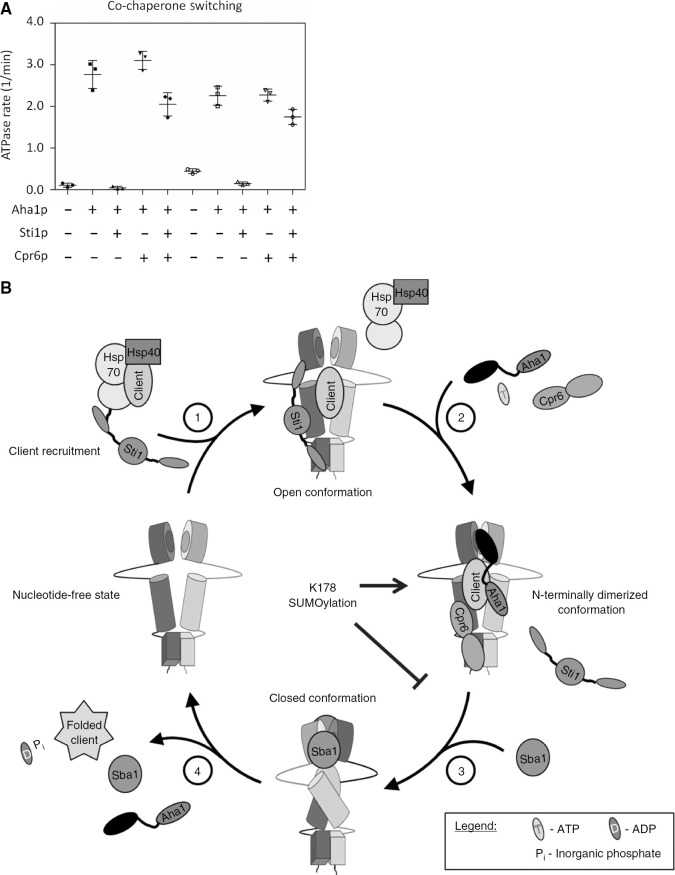
The cooperative displacement of Sti1p from SUMOylated Hsp82p and a model for SUMO regulation of the ATPase cycle. (A) Effective displacement of Sti1p by Cpr6p and Aha1p restore ATPase activity of SUMOylated Hsp82p^K178C^ to a similar degree as non-SUMOylated Hsp82p^K178C^. Reactions contained 1 μm Hsp82p^K178C^ (black) or Hsp82p^K178C^-Smt3p^Cys^ (gray) and 4 μm Sti1p, Cpr6p, and Aha1p where indicated. Each scatter plot shows the average and standard deviation from three triplicate experiments (n=3). (B) A model of the role SUMOylation has on its ATPase cycle of Hsp90. SUMOylation of yeast Hsp90 at K178 does not interfere with Sti1p displacement, but results in a stable recruitment of Aha1p which hinders Sba1p inhibition.

The Hsp90 client activation cycle involves the sequential recruitment and displacement of co-chaperones which is likely regulated by PTMs like SUMOylation. Sti1p is a potent inhibitor of ATPase activity because it enforces an ‘open’ conformation that is thought to define the beginning of the client activation cycle (Richter et al., 2003; Hessling et al., 2009). Aha1p and Cpr6p (a co-chaperone that, like Sti1p, binds to the *C* terminal MEEVD peptide in Hsp90) can cooperatively and potently displace Sti1p from Hsp90 (Li et al., 2013; Wolmarans et al., 2016). We wondered if SUMOylation would interfere with this ‘co-chaperone switching’ process. To examine this, we tested Aha1p and Cpr6p for the ability to overcome Sti1p inhibition of ATPase activity of SUMOylated and non-SUMOylated Hsp82p^K178C^. Aha1p and Cpr6p could overcome inhibition by Sti1p for both SUMOylated and non-SUMOylated Hsp82p^K178C^ (Figure 6A).

## Discussion

We report here a novel method for site-specific chemical SUMOylation of Hsp90 that recapitulates stable Aha1p recruitment *in vitro*. This method takes advantage of the fact that both Hsp82p and Smt3p naturally lack cysteine residues. It is important to note that this is not an uncommon trait: over 620 proteins in the yeast proteome (~10%) and over 560 proteins in the human proteome (~5%) lack cysteines. Even for proteins that do contain some cysteine residues (i.e. there are over 2000 proteins in the human proteome that contain two or fewer cysteines) it is possible that the introduction of cysteines at SUMOylation sites could result in preferential crosslinking. Under the conditions we describe, we achieve efficient crosslinking (~95%) that allows for the generation of large quantities of uniformly modified protein. This strategy overcomes the difficulties associated with *in vitro* SUMOylation using purified enzymes (i.e. lack of specificity) and isolation from cells (i.e. poor yield). Interestingly, ubiquitin also lacks cysteine residues and thus, our approach could theoretically be used for the *in vitro* site-specific modification of proteins with ubiquitin as well.

Hsp90 is vastly more abundant than the co-chaperone proteins that regulate the client activation cycle (Gasch et al., 2000; Ghaemmaghami et al., 2003). Therefore, controlling the temporal recruitment of co-chaperone proteins is of critical importance to the cellular function of Hsp90. The growing list of PTMs that have been detected in Hsp90 are increasingly being recognized as critical determinants of co-chaperone interaction. SUMOylation of the Hsp90 *N* domain is a conserved event and appears to regulate elements of Hsp90 function in cells (Mollapour et al., 2014). Preventing SUMOylation of Hsp82p at K178 does not have significant effects on client activation or Hsp90 function but enhanced SUMOylation provides clues regarding the significance of this modification. Enhancing SUMOylation promotes the degradation of CFTR and impairs v-src and glucocorticoid receptor (GR) activation. Enhancing SUMOylation of K178 in Hsp90 also appears to be sufficient to activate the heat shock response – presumably by diminishing the ability of Hsp90 to maintain HSF proteins in an inactive, unassembled state in the cytoplasm. It is not clear if all of these phenotypes are due solely to enhanced recruitment of Aha1p to SUMOylated Hsp90, but does suggest that Aha1p generally serves to diminish Hsp90 function in cells or can direct bound client proteins to degradation and quality control pathways (Wang et al., 2006).

SUMOylation of Hsp90 was reported to occur only on one subunit of the dimer (Mollapour et al., 2014). Our results suggest that the asymmetry observed *in vivo* may be due to the low frequency of SUMOylation of target proteins, even when enhanced by SUMO overexpression, rather than any functional impediment associated with SUMOylation on both sides of the dimer. Indeed, the intrinsic and Aha1p-stimulated ATPase rates of Hsp82p^K178C^ were very similar regardless of whether or not Smt3p^Cys^ had been crosslinked to one or both subunits. Hsp90 may have evolved such that the dimer can respond to a SUMOylation event on only one subunit for functional efficiency. Indeed, this makes sense given the vast abundance of Hsp90 in relation to co-chaperones and enzymes that modify Hsp90. Alternatively, it is also possible that SUMOylation of one subunit blocks the interaction with the SUMOylation machinery that would be required for modification of the other subunit.

The modification strategy we describe herein appears to weakly favor the *N* terminally dimerized conformation of Hsp90. This is consistent with the fact that both the affinity for Aha1p on its own as well as the intrinsic ATPase rate is increased by modification with the BMOE crosslinker – events that are both known to occur when *N* terminal dimerization is enhanced (Retzlaff et al., 2010; Pullen and Bolon, 2011). The hydrophobic BMOE crosslinker could be directly enhancing *N* terminal dimerization in a manner similar to hydrophobic amino acid substitutions in this area or indirectly by favoring lid closing, which exposes hydrophobic interaction surfaces between the *N* domains (Hawle et al., 2006; Vaughan et al., 2009). Interestingly, the apparent affinity of Sba1p for Hsp82p in the presence of Aha1p was unchanged by the BMOE derivatization. Thus, the reduced affinity of Sba1p for SUMOylated Hsp82p we observed in the presence of Aha1p is due to the presence of the crosslinked SUMO group and not the BMOE crosslinker.

Our work here highlights the general question of how PTMs like SUMOylation of K178, and phosphorylation of T22 and Y24, can all apparently be required for stable Aha1-Hsp90 interaction in cells when these two proteins readily interact *in vitro* in the absence of modifications. It seems likely that these PTMs trigger a cascade of events, potentially including the addition or removal of other PTMs or recruitment of other factors, which result in far greater changes in affinity. However, in our defined system, we can assess the individual contribution of SUMOylation of K178 on Aha1p binding as well as effects on enzymatic activity. Conjugation of Smt3p^Cys^ at position 178 likely results in the adoption of an Hsp90 conformation from which Aha1p is more difficult to dislodge by Sba1p. It is interesting to consider that Aha1 overexpression, p23 silencing (the human homologue of Sba1p), or enhanced Hsp90 SUMOylation all decrease the stability of CFTR (Wang et al., 2006; Mollapour et al., 2014). Taken together, this suggests that SUMOylation of Hsp82p at K178 stalls the Hsp90 cycle at a point after Aha1 binding but before Sba1p binding (Figure 6B). Indeed, PTMs of Hsp90 may serve to ‘stall’ the cycle at various stages to modulate client protein folding. We find that binding of Sti1p is unperturbed, likely because Sti1p is thought to act before Aha1p and binds primarily to the MEEVD peptide at the *C* terminus of Hsp90 and forces the chaperone into the ‘open’ conformation (Rohl et al., 2015). The model we propose here is in contrast to other protein-protein interactions that are mediated by SUMOylation that involve SUMO interaction motifs (SIMs) (Hannich et al., 2005; Hecker et al., 2006). An analysis of the Aha1p primary sequence does not reveal any SIMs and we did not detect any co-immunoprecipitation of monomers or crosslinked dimers of Smt3p with Aha1p, or any other co-chaperone we tested, in our experiments. It seems that the effect of SUMOylation is linked to alterations in the conformational dynamics of Hsp90 and not to specific recruitment of co-chaperones.

**Figure 7:**
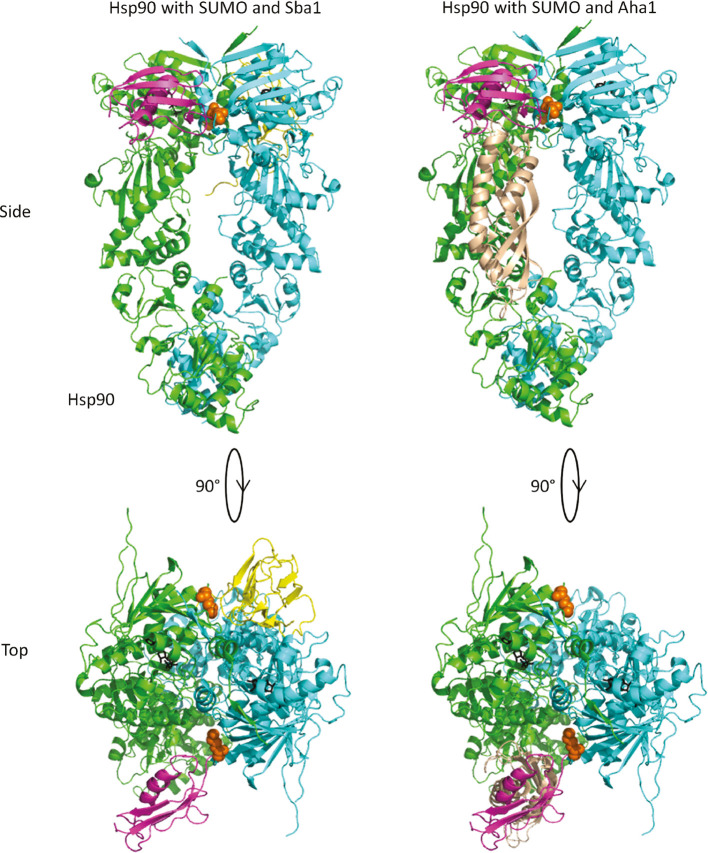
A model for Sba1p and Aha1p binding to SUMOylated Hsp82p. The Hsp82p dimer is shown in green and cyan. Lysine 178 is shown in orange spheres. Sba1p and Aha1p (N-domain only from PDBID 1USV; Meyer et al., 2004) are shown in yellow and beige, respectively. Smt3p is shown in magenta and modeled near lysine 178 (Sheng and Liao, 2002). The left panel shows that Sba1p and Smt3p coupled to position 178 of Hsp82p could occupy a similar space (each shown on the opposite side of the dimer). The right panel shows the position of the Aha1p *N* domain near where Smt3p would be coupled to lysine 178. PDBIDs 2CG9, 1L2N, 1USV (Sheng and Liao, 2002; Meyer et al., 2004; Ali et al., 2006).

The location of lysine 178 is near the interface of the dimerized *N* domains (Figure 7, orange sphere representation). This could potentially block or interfere with binding of either Sba1p (Figure 7, left panel) or the *C* terminal domain of Aha1p (Figure 7, right panel) (Ali et al., 2006; Martinez-Yamout et al., 2006; Koulov et al., 2010; Retzlaff et al., 2010). However, SUMO proteins have long, flexible *C* terminal linkers separating the globular region of the protein from the attachment site (Sheng and Liao, 2002). This allows for the SUMO protein to occupy many different places near the target site without being rigidly held in place. Our data suggests that the crosslinked Smt3p on its own does not block the binding of either Sba1p or Aha1p because each co-chaperone interacted robustly with SUMOylated Hsp82p in our IP experiments. Moreover, the Aha1p *C* terminus was able to participate in ATPase stimulation in our ATPase assays with SUMOylated Hsp82p. That Sba1p was far less able to inhibit Aha1p-mediated ATPase stimulation of SUMOylated Hsp82p compared to wildtype Hsp82p suggests that the Sba1p binding interface could be less accessible when both the Aha1p C-terminus and Smt3p are present. Crowding of the shared binding interface of Sba1p and the Aha1p *C* domain by SUMOylation of lysine 178 could prevent or alter competition between these two co-chaperones *in vivo*.

It is important to acknowledge that SUMOylation is normally accomplished through the formation of an isopeptide bond which is slightly shorter, and less bulky, than the homobifunctional maleimide crosslinker we use in this study. However, our method recapitulates *in vivo* observations suggesting that we are at least partially capturing the real effects of SUMOylation.

There are hundreds of PTMs that have been detected in Hsp90 and its many co-chaperones. The painstaking process of integrating all of them into what could potentially be numerous distinct client activation cycles requires the ability to isolate individual events for study. Our novel strategy provides a means of generating site-specific and efficient modification of proteins with SUMO for enzymatic, biochemical, or structural analysis.

## Materials and methods

### Cloning

Site-directed mutagenesis was carried out to construct Hsp82p^K178C^ using QuikChange mutagenesis (Agilent, Santa Clara, CA, USA). The coding sequence of Hsp82p^K178C^ was verified by sequencing. Hsp82p^K178C^ was sub-cloned into pET11dHisMyc and pET11dHisFlag vectors as previously described, where the Myc and Flag epitope was fused in-frame with the 6xHis-tag sequence upstream of the *Nde*I site of the pET11dHis vector (Wolmarans et al., 2016). The SMT3 coding sequencing was amplified by polymerase chain reaction (PCR) with primers designed to introduce *Nde*I and *Bam*HI restriction sites at the 5′ and 3′ ends, respectively. The mature form of Smt3p was generated by replacing the C-terminal region (amino acids 98-101 or 99-101) with a cysteine residue using the according primers (sense: 5′-gagagacatatgtcggactcagaagtcaatcaagaagc-3′ and antisense: 5′-ctctcggatcctcactagcaaccaccaatctgttctctgtgag-3′ or 5′-tctctcggatcctcactagcaaccaatctgttctctgtgag-3′). The PCR product was digested with *Nde*I and *Bam*HI for ligation into similarly cut pET11dHis vector.

### Plasmid expression

Protein expression and purification of *Saccharomyces cerevisiae* proteins was carried out as previously described (Armstrong et al., 2012; Horvat et al., 2014; Wolmarans et al., 2016). Vectors encoding Hsp82p, Hsp82p^K178C^, Smt3p^Cys^, Aha1p, Sti1p, Cpr6p, Sba1p, and Hch1p were expressed in *Escherichia coli* strain BL21 (DE3) from pET11dHis, pET11dHisMyc, or pET11dHisFlag (Stratagene, La Jolla, CA, USA). Cells were grown to an OD600 of 1.0, induced with 1 mm isopropyl-1-thio-D-galactopyranoside (IPTG), and incubated 37°C (Smt3p^Cys^, Aha1p, Sti1p, Cpr6p, and Sba1p) or 30°C (Hsp82p, Hsp82p^K178C^, and Hch1p) for expression. Cultures were harvested after overnight growth, except for Hch1p that was harvested after 8 h of growth. Bacterial pellets were lysed as previously described (Armstrong et al., 2012; Horvat et al., 2014; Wolmarans et al., 2016). The final step of Hsp82p^K178C^ and Smt3p^Cys^ purification (size exclusion chromatography) was conducted in gel filtration buffer free of reducing agents (25 mm Hepes pH 7.2, 10 mm NaCl for Hsp82p^K178C^ and 25 mm Hepes pH 7.2, 50 mm NaCl for Smt3p^Cys^).

### Crosslinking of Hsp82p^K178C^ to Smt3p^Cys^

To ensure crosslinking of Hsp82p^K178C^ to Smt3p^Cys^ and not to itself, 32 μm Hsp82p^K178C^ is derivatized first by incubating it with 200 μm BMOE for 1 h at room temperature. Smt3p^Cys^ is then titrated (added, mixed, and incubated at room temperature for another hour) into derivatized Hsp82p^K178C^ to establish at which concentration of Smt3p^Cys^ resulted in the most crosslinked product. From the crosslinking experiments shown in Figure 2, it was established that optimal crosslinking occurred when 250 μm of Smt3p^Cys^ was added to 2 μm of derivatized Hsp82p^K178C^. Using this ratio (250 μm of Smt3p^Cys^ to every 2 μm of Hsp82p^K178C^), Smt3p^Cys^ was added to non-derivatized Hsp82p^K178C^, derivatized Hsp82p^K178C^, or derivatized and DTT-quenched Hsp82p^K178C^ reactions for experiments shown in Figures 3, 4C, 5, and 6A. A specified concentration of Smt3p^Cys^ was added into derivatized and non-derivatized Hsp82p^K178C^ reactions in Figures 2C and 4A,B. To stop the crosslinking reaction (prior to the addition of Smt3p^Cys^ into the ‘derivatized but non-SUMOylated’ Hsp82p reactions in Figures 3 and 4C) and to quench all unreacted reactive groups prior to the addition of co-chaperones and other ATPase components, 30 mm DTT is added and incubated for 15 min (except for Figure 4C, to which only 10 mm DTT was added). Crosslinking efficiency was assessed by running a sample of the derivatized and crosslinked reaction on an 8% and 12% SDS-PAGE gel stained with Coomassie blue. Control reactions used in experiments have DMSO present, instead of BMOE.

### Crosslinking under different nucleotide conditions

We utilized two approaches to test the efficacy of chemically SUMOylating Hsp82p^K178C^ under different nucleotide conditions. In a titrating experiment (Supplementary Figure 1A), 32 μm Hsp82p^K178C^ was either pre-incubated with 5 mm ADP, AMPPnP, or control buffer (100 mm Tris pH 7.4) conditions for 5 min. After nucleotide binding dictated Hsp82p^K178C^ to adopt specific conformations, 200 μm BMOE and was incubated for 10 min. Crosslinking reactions were set up by adding 2 μm of the derivatized Hsp82p^K178C^ (Hsp82p^K178C^*) to tubes also containing 5 mm ADP, AMPPNP, or control buffer with either 0, 20, 60, or 120 μm Smt3p^Cys^ and incubated for 20 min. Reactions were then quenched with 30 mm DTT for 5 min. Crosslinking efficiency was assessed by running a sample of each reaction on a 10% SDS-PAGE gel, followed by Coomassie blue staining. A timepoint assay (Supplementary Figure 2A) was also conducted to investigate how nucleotide conditions influence the rate of chemical SUMOylation. In this experiment, 32 μm Hsp82p^K178C^ was first pre-incubated with 5 mm ADP, AMPPnP, or control buffer conditions for 2 min, followed by the addition of 200 μm BMOE for a 10-min incubation. A total of 2 μm of the derivatized Hsp82p^K178C^ was then aliquoted into reactions containing 5 mm ADP, AMPPNP, or control buffer in the presence of either 0 μm Smt3p^Cys^ (control – lane 1) or 100 μm Smt3p^Cys^ (lanes 2, 3, 4). Reactions were quenched at 0, 5, 10 and 20 min with 30 mm DTT for 5 min. Crosslinking rate was assessed by running a 10 μl sample of each reaction on a 10% SDS-PAGE gel, followed by Coomassie blue staining.

### Data analysis of crosslinking reactions under different conditions

The band intensities of Hsp82p^K178C^* and Hsp82p^K178C^-Smt3p^Cys^ were measured from the stained gels from each experiment (shown in Supplementary Figures 1A and 2A) using multiplex band analysis function on AlphaView software (FluorochemQ, Protein Simple). To calculate the percent of SUMOylated Hsp82p^K178C^ in each reaction, the background noise was first subtracted from all reactions. Secondly, the intensity of the SUMOylated band (Hsp82p^K178C^-Smt3p^Cys^) was divided by the sum of the SUMOylated and the non-SUMOylated band intensities (Hsp82p^K178C^-Smt3p^Cys^+Hsp82p^K178C^*) in each reaction. The average was then calculated from the three independent experiments and expressed as a percent. Using Prism GraphPad, the percentage of the crosslinked product from the titration experiment was plotted in a bar graph with the standard error of the mean (Supplementary Figure 1B). The percentage of the crosslinked product from the timepoint experiment was plotted in a XY plot as a function of time and a fit-lines calculated on Prism GraphPad (Supplementary Figure 2B). One-way ANOVA analysis was also performed from the individual replicates (n=3) of both the titration and timepoint experiments using Prism GraphPad and Tukey’s *post-hoc* analysis.

### ATPase assays

All ATPase assays were carried out using the PK/LDH regenerating system as previously described (Panaretou et al., 1998; Armstrong et al., 2012; Horvat et al., 2014; Wolmarans et al., 2016). Average values of the experiments are shown with error expressed as standard deviation using Prism GraphPad. The ATPase rates are either shown as μm ATP hydrolyzed per minute per μm of Hsp82p (1/min) or as a percentage of the maximally Aha1p-stimulated ATPase rate of Hsp82p, Hsp82p^K178C^, Hsp82p^K178C*^ (derivatized), or Hsp82p^K178C^-Smt3p^Cys^. Fit lines were calculated according to the following equation: (Y=((B_max_*X)/(*K*_app_+X))+X0). The final conditions of all the reactions are 25 mm Hepes (pH 7.2), between 1 and 25 mm NaCl, 5 mm MgCl_2_, 1–2 mm DTT, 0.3 mm NADH, 2 mm ATP, 1 mm phosphoenol pyruvate (PEP), 2.5 μl of pyruvate kinase/lactate dehydrogenase (PK/LDH) (Sigma), and 0.5% DMSO. Identical reactions were quenched with 50–100 μm NVP-AUY922 and subtracted from unquenched reactions to correct for contaminating ATPase activity.

### Immunoprecipitation assay

*In vitro* immunoprecipitation assays were conducted using Ultralink Protein G beads (Pierce Thermo Fisher) that had been coupled to anti-myc monoclonal antibodies (clone 9E10) at a concentration of 5 μg antibody per 1 μl of beads. To assess co-chaperone binding to SUMOylated or non-SUMOylated Hsp82p^K178C^, 5 μm of 6×HisMyc-tagged Aha1p, Sti1p, Cpr6p, Sba1p, or Hch1p was mixed with equimolar 6xHis-tagged Hsp82p^K178C^ or Hsp82p^K178C^-Smt3p^Cys^ in the presence of 5 mm AMPPnP or ADP. Samples containing crosslinker BMOE was treated with 10 mm DTT and incubated for 30 min to quench all reactive groups before adding 6×HisMyc-tagged co-chaperones. Final buffer conditions of all reactions were 25 mm Hepes (pH 7.2), 10–15 mm NaCl, 5 mm MgCl_2_, 0.1% tween-20, 1 mm AMPPnP or ADP, 1.0–1.5 mm DTT, 0.01 μl HALT protease inhibitor. Ten microliter of Ultralink Protein G beads coupled to anti-Myc monoclonal antibodies were added to all these 50 μl reactions followed by a room temperature incubation on a rotator for 60 min. Beads were then pelleted, washed once in 250 μl of binding buffer, resuspended in 50 μl SDS sample buffer, and run on SDS-PAGE. Complexes were analyzed by Coomassie blue staining.

## Supplementary Material

Supplementary material
