# Supplementary material for: A novel method for site-specific chemical SUMOylation: SUMOylation of Hsp90 modulates co-chaperone binding in vitro
Source: Biol Chem. 2018 Oct 12;400(4):487–500. doi: 10.1515/hsz-2018-0251 (PMC9605714; doi:10.1515/hsz-2018-0251)
Supplement: Supplementary file 1 — Supplementary material [file j_hsz-2018-0251_suppl.pdf]

A novel method for site-specific chemical SUMOylation: SUMOylation of Hsp90 modulates co-chaperone binding *in vitro*

Annemarie Wolmarans, Allison Kwantes and Paul LaPointe

Supplementary material

Supplemental
Figure 1a

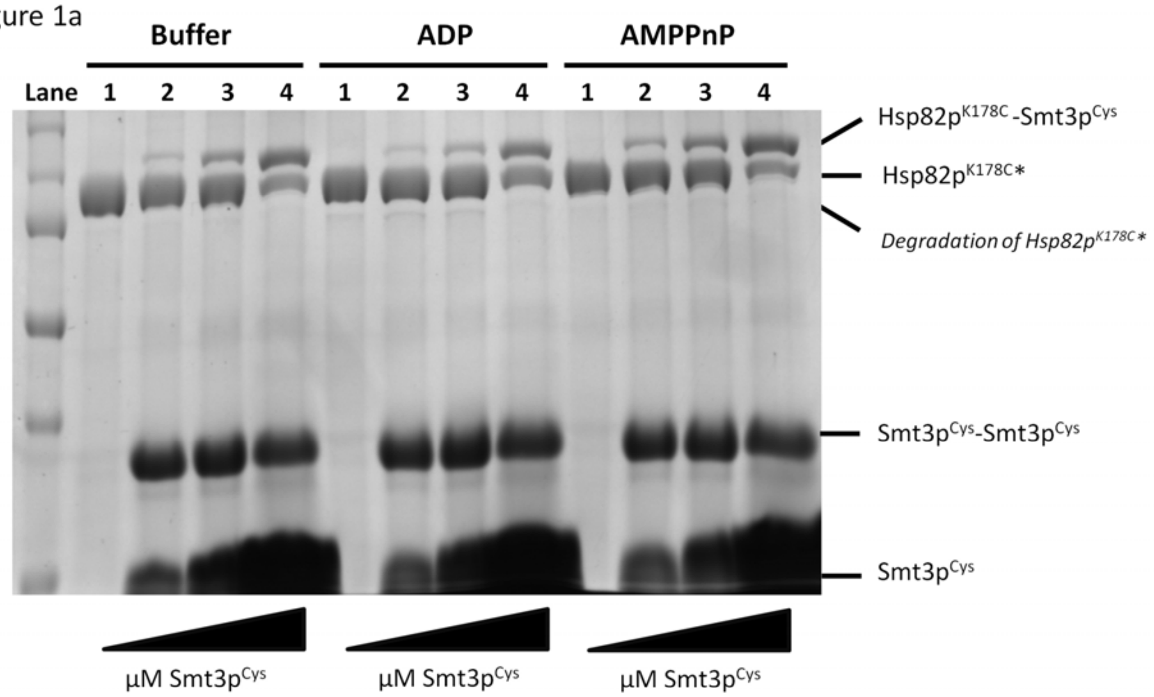

Supplemental
Figure 1b

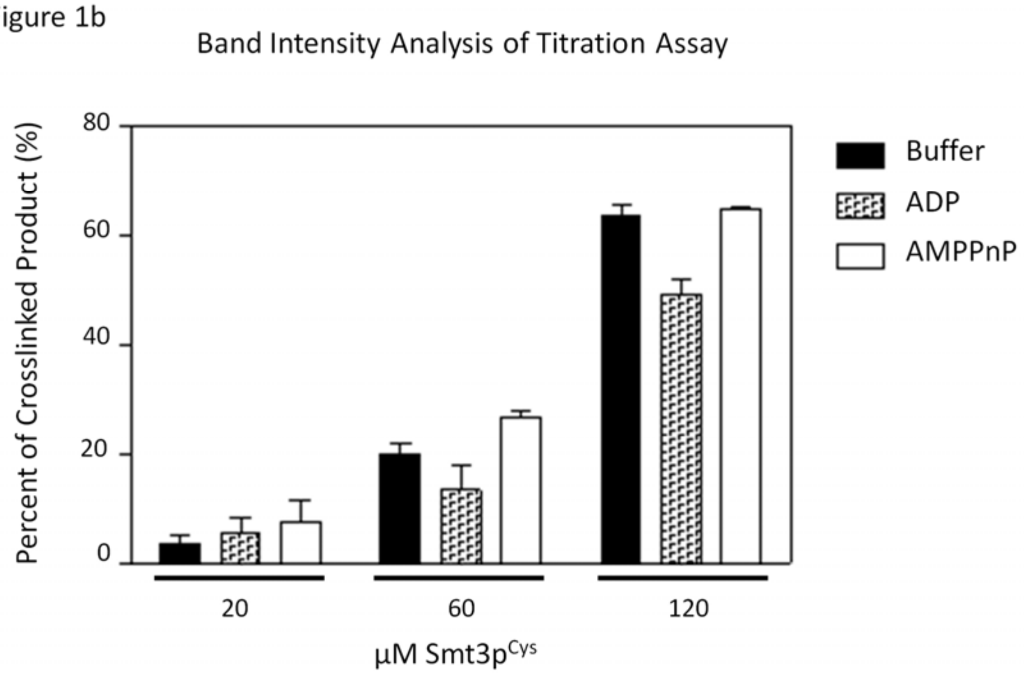

Supplementary Figure 1 Hsp82p^{K178C} derivatization and conjugation to Smt3p^{Cys} performed under different nucleotide conditions in a titration assay.

A. Less SUMOylated product is produced in ADP conditions compared to buffer or AMPPnP conditions. Each reaction contains 2 μM Hsp82p^{K178C}*, 5 mM nucleotide (buffer, ADP, or AMPPnP), and the indicated amount of Smt3p^{Cys} (0, 20, 60, or 120 μM). A representative gel of the titration experiments is shown. **B.** Band intensity analysis of titration experiments show there is no statistically significant difference in the percent of crosslinked product (Hsp82p^{K178C}-Smt3p^{Cys}) between the three conditions. Band intensities were measured of crosslinking reactions shown in (A), analyzed as described in methods, and results plotted in a bar graph. The average percent of crosslinked product with the standard error of the mean for buffer (black), ADP (grey), and AMPPnP (white) conditions are shown from three experiments (n=3).

Supplemental Figure 2a

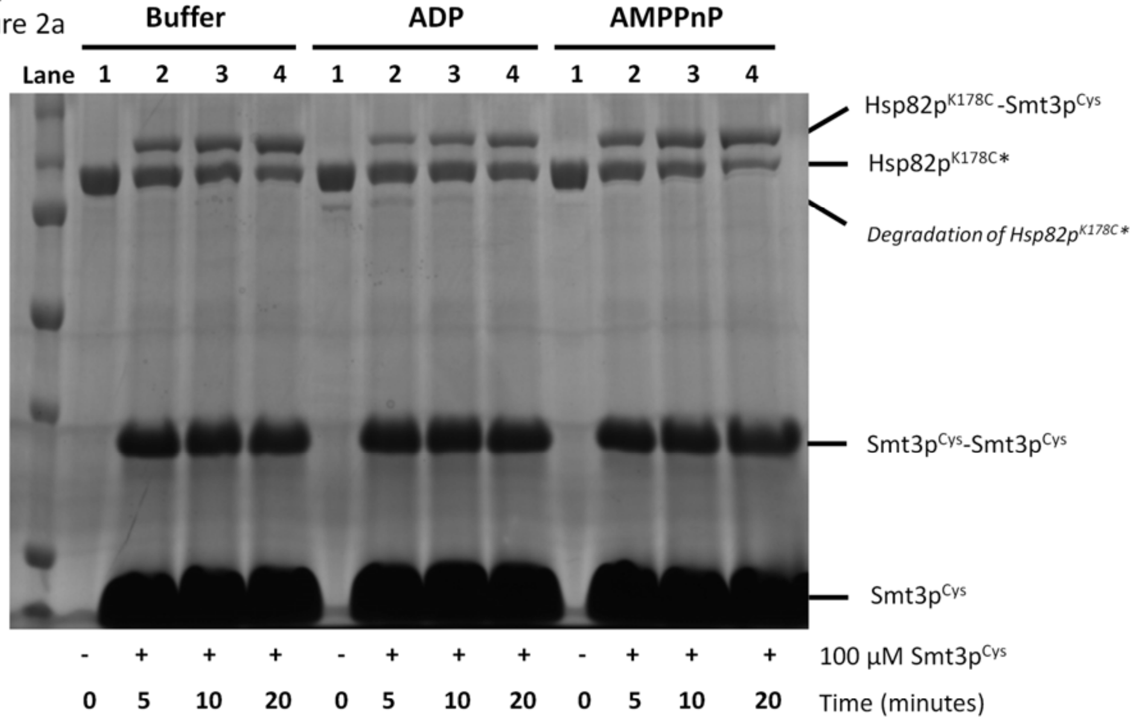

Supplemental Figure 2b

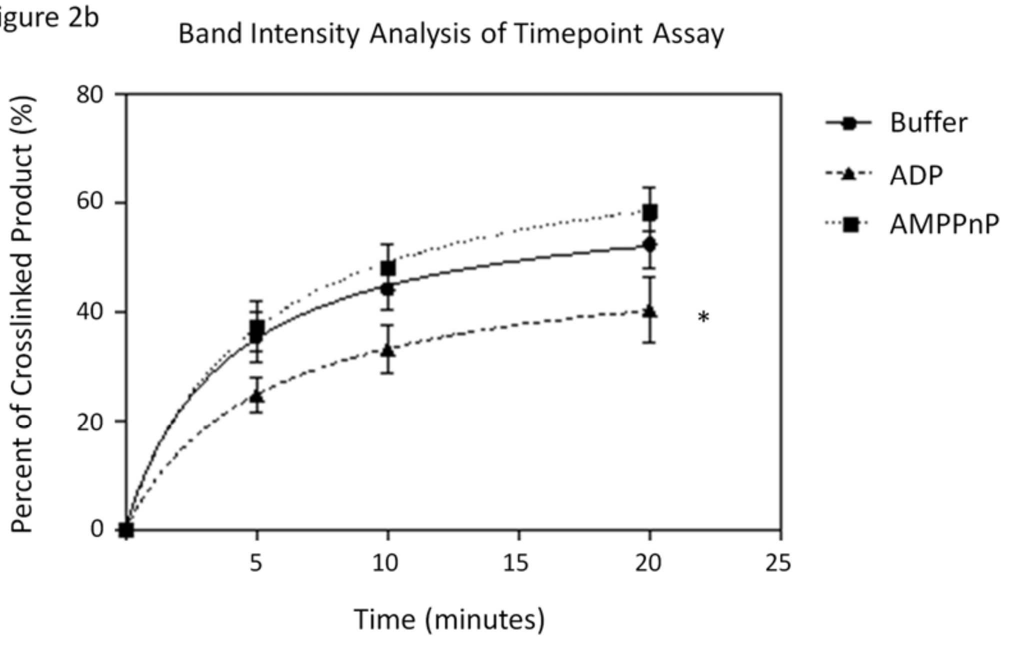

Supplementary Figure 2 Hsp82p^{K178C} derivatization and conjugation to Smt3p^{Cys} performed under different nucleotide conditions in a timepoint assay.

A. ADP conditions results in a slower rate of chemical SUMOylation. Each reaction contains 2 µM Hsp82p^{K178C}*, 5 mM nucleotide (buffer, ADP, or AMPPnP), and either 0 or 100 µM Smt3p^{Cys}. Reactions were stopped at indicated timepoints by quenching the reaction with 30 mM DTT. A representative gel of the timepoint experiments is shown. **B.** Band intensity analysis of timepoint experiments show ADP conditions results in a statistically significant decrease in the percent of crosslinked product (Hsp82p^{K178C}-Smt3p^{Cys}) compared to the buffer and AMPPnP conditions (* - one-way ANOVA, $p < 0.05$). Band intensities were measured of crosslinking reactions shown in (A), analyzed as described in methods, and results plotted in a XY plot. The average percent of crosslinked product with the standard error of the mean for buffer (circles; solid line), ADP (triangles; dashed line), and AMPPnP (squares; stippled line) conditions are shown from three experiments (n=3).
